# Antioxidant supplementation may effect DNA methylation patterns, apoptosis, and ROS levels in developing mouse embryos

**DOI:** 10.1007/s00418-024-02286-w

**Published:** 2024-04-17

**Authors:** Fatma Uysal, Gozde Sukur, Nazlican Bozdemir, Ozgur Cinar

**Affiliations:** 1 Department of Histology and Embryology, Ankara Medipol University School of Medicine, Altindag, 06050 Ankara, Turkey; 2 Department of Histology and Embryology, Ankara University School of Medicine, Altindag, 06080 Ankara, Turkey; 3 Department of Molecular Biology and Genetics, Gebze Technical University, Kocaeli, Turkey

**Keywords:** Antioxidants, Blastocyst, DNA methylation, Embryo culture, Oxidative stress

## Abstract

This study was designed to address the question: does antioxidant-containing embryo culture media affect DNA methyltransferases, global DNA methylation, inner cell mass/trophoblast differentiation, intracellular reactive oxygen species (ROS) levels, and apoptosis? Mouse zygotes were cultured in embryo culture media containing MitoQ, *N*-acetyl-l-cysteine (NAC), acetyl-l-carnitine (ALC), α-lipoic acid (ALA), or the mixture of NAC + ALC + ALA (AO) until the blastocyst stage, whereas in vivo-developed blastocysts were used as control. Protein expression levels of Dnmt1, 3a, 3b, and 3l enzymes were analyzed by immunofluorescence and western blot, while global DNA methylation, apoptosis, and ROS levels were evaluated by immunofluorescence. NAC, ALC, and MitoQ significantly increased the levels of all Dnmts and global methylation. ALA significantly induced all Dnmts, whereas global methylation did not show any difference. NAC and mixture AO applications significantly induced Nanog levels, ALA and MitoQ increased Cdx2 levels, while the other groups were similar. ALA and MitoQ decreased while ALC increased the levels of intracellular ROS. This study illustrates that antioxidants, operating through distinct pathways, have varying impacts on DNA methylation levels and cell differentiation in mouse embryos. Further investigations are warranted to assess the implications of these alterations on the subsequent offspring.

## Introduction

In vitro culture systems have long been employed for developing embryos in infertility treatment. Nevertheless, the development of embryos in vitro is adversely influenced by various conditions, including oxidative stress (Agarwal et al. 2022). Reactive oxygen species (ROS) produced in vitro delay embryo development and therefore may reduce in vitro fertilization (IVF) success. Embryo culture in low oxygen conditions and/or with antioxidant supplementation have been proposed to protect the embryos from ROS effects (Agarwal et al. 2022; Hardy et al. 2021).

The utilization of different antioxidants acting via different pathways has been proposed and now, antioxidants containing culture media have been used in embryology laboratories. The beneficial effects of *N*-acetyl-l-cysteine (NAC), acetyl-l-carnitine (ALC), and α-lipoic acid (ALA) on embryo development have previously been documented (Dunning and Robker 2012; Abdelrazik et al. 2009; Selvakumar et al. 2006; Zhang et al. 2013; Linck et al. 2007). We recently showed that exposure of GV-stage oocytes to mitochondria-targeted antioxidant, MitoQ, protected against spindle and chromosomal defects in mouse oocytes exposed to oxidative stress or obtained from reproductively aged mice (Al-Zubaidi et al. 2021). Additionally, MitoQ promoted nuclear maturation and protected against chromosomal misalignments in human oocytes. *N*-acetyl-l-cysteine (NAC) is a precursor of reduced glutathione (GSH), which plays a critical role in protecting cells from oxidative damage (Meister and Tate 1976; Meister and Anderson 1983; Hammond et al. 2001). ALC protects against oxidative stress through scavenging free radicals (Gülçin 2006). ALC supplementation of mouse embryo culture media containing hydrogen peroxide, an exogenous oxidative stress promoter, improves oocyte chromosomal structure and blastocyst development, and reduces DNA damage (Abdelrazik et al. 2009). ALA acts as a potent free radical scavenger and metal chelator and is responsible for recycling other cellular antioxidants including glutathione (GSH) and vitamins C and E (Packer et al. 1995; Bilska and Włodek 2005). The addition of ALA to mouse embryo culture media improves embryo development at high oxygen tension by protecting the embryos against oxidative stress (Linck et al. 2007).

Epigenetic mechanisms are one of the fundamental regulators of gene expression without changing the primary nucleotide sequence. One of the epigenetic mechanisms is DNA methylation, which is controlled by DNA methyltransferases (DNMTs), and it controls the functions of genes related to cell survival, death, differentiation, cycle, and autophagy, and therefore may affect accurate embryo development. Briefly, Dnmt1 is responsible for the maintenance of methylation by transferring methyl groups to the hemi-methylated DNA strands following DNA replication. Among the three Dnmt3 proteins, Dnmt3a and Dnmt3b are essential for de novo methylation, and Dnmt3l indirectly contributes to the de novo methylation process (Okano et al. 1999). All these Dnmts are crucial for preimplantation embryo development (Uysal et al. 2015). We recently demonstrated that a sequential or single-step embryo culture media approach for in vitro embryo development altered DNMTs and global DNA methylation by indicating that the composition of embryo culture media differentially affects Dnmts and global DNA methylation (Uysal et al. 2021).

In the current study, we evaluated the effects of different antioxidants added in embryo culture media on DNMTs, global DNA methylation, and ROS levels, during in vitro mouse preimplantation embryo development. Moreover, as oxidative stress or antioxidant treatment can alter the expression of pluripotency-related genes/proteins (Nanog and Cdx2) and cell survival (Geng et al. 2023; Li et al. 2022; Yu et al. 2023), we also analyzed embryo/trophoblast cell differentiation and apoptosis.

## Materials and methods

### Animals

The experimental protocol was approved by the Animal Care and Usage Committee of Ankara University (protocol no. 2022-12-101). Female Balb/C mice at 4–6 weeks and male mice at 8–10 weeks of age were purchased from the Research Animal Laboratory Unit. All mice were hosted with free access to food and water and kept in a 12 h light/dark cycle.

### Collection of zygotes and in vitro embryo culture

To collect zygotes, intraperitoneally 5 IU pregnant mare’s serum gonadotropin (PMSG) (Intervet, Türkiye), and 48 h later 5 IU human chorionic gonadotropin (hCG) (Sigma-Aldrich, USA)-injected female mice were kept with mature male mice at a rate of one female:one male overnight for mating. The presence of vaginal plug verified the fertilization and zygotes were obtained from the oviducts of sacrificed pregnant female mice at 20 h following hCG injection. The cumulus cells surrounding the zygotes were removed using hyaluronidase (Vitrolife, Sweden) solution at a concentration of 5 IU/mL. Embryos were immediately placed in morpholinepropanesulfonic acid (MOPS)-buffered medium (G-MOPS) (Vitrolife, Sweden) after collection and then transferred to embryo culture medium (G-TL; Vitrolife, Sweden) as 30 μL volumes of culture drops in 35 mm culture dishes (Corning, USA) that were overlaid by approximately 3 mL of paraffin oil (OVOIL; Vitrolife, Sweden). Zygotes (0 h) were randomly cultured in the related culture media to the blastocyst stage (96 h) at 37 °C in 6% CO_2_.

For the in vivo control group, pregnant females were allocated for 96 h, and after sacrificing, blastocysts were collected from the uterus. Three hundred embryos were evaluated for each group. All experiments were performed in at least three replicates.

### Experiment groups

The groups were designed as follows:

1. In vivo control group: Blastocysts developed in vivo were collected from mice.
2. In vitro control group: Embryos were cultured in a medium without antioxidants.
3. NAC group: Embryos were cultured in 10 µM of NAC-containing culture medium (Truong et al. 2016)
4. ALC group: Embryos were cultured in 10 µM of ALC-containing culture medium (Truong et al. 2016)
5. ALA group: Embryos were cultured in 5 µM of ALA-containing culture medium (Truong et al. 2016)
6. MitoQ group: Embryos were cultured in 50 nM of MitoQ-containing culture medium (Al-Zubaidi et al. 2021)
7. The mixture AO group: Embryos were cultured in a medium containing a combination of 10 µM of NAC, 10 µM of ALC, and 5 µM of ALA.

### Immunofluorescence (IF) staining

Blastocysts were fixed and then permeabilized with 4% paraformaldehyde (Sigma-Aldrich, USA) solution and 1% Tween-20 (Sigma-Aldrich, USA) at room temperature (RT), respectively. After blocking in 20% normal goat serum-containing solution (Vector Laboratory, USA), IF was applied to detect the relative signal intensities and cellular distribution profiles of the Dnmt1, Dnmt3a, Dnmt3b, Dnmt3L, Cdx2, and Nanog proteins in the blastocysts. Briefly, blastocysts were incubated overnight at +4 °C with the primary antibodies against Dnmt1 (1:100; ab87654, Abcam; UK), Dnmt3a (1:100; ab188470, Abcam, UK), Dnmt3b (1:100; 48,488, Cell Signaling, USA), Dnmt3L (1:100; ab3493, Abcam, UK), 5mC (1:200; 28,692, Cell Signaling, USA), Cdx2 (1:100; ab76541, Abcam, UK), or Nanog (1:100; 8822, Cell Signaling, USA). After a triple wash with 1× phosphate buffered saline solution including 2% bovine serum albumin (BSA) for 10 min each (PBS–BSA; Sigma-Aldrich, USA), blastocysts were incubated with anti-rabbit IgG Alexa 488-conjugated secondary antibody (Invitrogen, USA) for 1 h at RT followed by triple washes with 1× PBS–BSA solution for 10 min each. The omission of primary antibodies served as a negative control. All staining steps were performed using mini-well trays (Thermo Fisher Scientific, USA) in a humidified chamber. Stained blastocysts were gently transferred onto glass-bottomed 35 mm Petri dishes in a 4 μL drop of PBS-based mounting medium containing 1 μg/mL Hoechst 33,342 (Thermo Fisher Scientific, USA) for DNA labeling. The top was covered with a droplet of paraffin oil (Ovoil, Vitrolife, Sweden). All fluorescently tagged specimens were examined and imaged using a Zeiss LSM-880 Airyscan system (Zeiss, Germany) with a 40× Zeiss C-Apo water immersion objective (1.2 NA). Alexa-488 was excited using the 488 nm laser and a band pass of green-fluorescent emission was 493–634 nm. Hoechst 33,342 was excited with 405 nm laser and emission was collected with a band pass of 438–458 nm. Images were captured with the Zeiss ZEN Black software. All laser power, pinhole, and gain parameters were set based on the negative control and were kept the same for each experiment.

### ROS level assay

Reactive oxygen species levels in blastocysts were determined with 2′,7′–dichlorofluorescein diacetate (DCFDA) Cellular ROS Assay Kit/Reactive Oxygen Species Assay Kit (ab113851, Abcam, UK). Live blastocysts were washed twice with 1× buffer (ab113851, Abcam, UK). After washing, the blastocyts were stained with 20 μM DCFDA at 37 ℃ for 45 min in the dark. Blastocysts were washed with 1× buffer twice. Then, blastocysts were placed in a 20 μL drop of 1× buffer covered with Ovoil (10,029, Vitrolife, Sweden). ROS levels were analyzed with a Zeiss LSM-880 Airyscan system.

### Terminal deoxynucleotidyl transferase dUTP nick end labeling (TUNEL) assay

The TUNEL assay was performed using the In Situ Cell Death Detection Kit (Sigma-Aldrich, USA) according to the manufacturer’s instructions. Following fixation of blastocysts in 4% paraformaldehyde and then permeabilization with 1% Tween-20 (Sigma-Aldrich, USA) at RT, embryos were washed three times in PBS. Embryos were incubated with TUNEL reaction mixture for 1 h at 37 ℃ in the dark. For the negative control, the enzyme solution was omitted. Blastocysts were gently transferred onto glass-bottomed 35 mm dishes in a 4 μL drop of PBS-based mounting medium containing 1 μg/mL Hoechst 33,342 (Thermo Fisher Scientific, USA). The top was covered with a droplet of paraffin oil. All fluorescently tagged blastocysts were examined and imaged using a Zeiss LSM-880 Airyscan system. The total number of nuclei and number of TUNEL-labeled nuclei were determined under microscope for each embryo. The ratio of TUNEL-positive cells to the total number of cells was defined as the apoptosis index. TUNEL staining for each group was performed at least three times.

### Western blotting (WB)

Semi-quantitative analysis of Dnmt1, Dnmt3a, Dnmt3b, and Dnmt3l was performed with western blotting (WB). For each group, embryos (*n* = 200) were placed in a lysis buffer (1% sodium dodecyl-sulfate, 1 mmol/L sodium ortho-vanadate, 10 mmol/L Tris pH 7.4) supplemented with 1× protease inhibitor cocktail (Amresco, USA). The protein concentration was measured using the bicinchoninic acid (BCA) method. Fifty micrograms of protein from each group were loaded on each lane of 10% SDS–polyacrylamide gel electrophoresis (PAGE) gel, which was used for protein electrophoresis Following electro-transfer to a polyvinylidene difluoride (PVDF) membrane (Roche, UK) overnight at +4 ℃, the membrane was blocked with 5% (w/v) BSA prepared in TBS-T (20 mmol/L Tris/HCl and 150 mmol/L NaCl plus 0.05% Tween-20 at pH 7.4) at RT for 1 h. Membranes were incubated with primary antibodies specific to Dnmts or β-actin (Abcam, USA) (1:1000 in 5% (w/v) BSA-containing TBS-T) for 2 h at RT. Following a triple-wash in TBS-T for 15 min each, membranes were incubated with IRDye 800CW Goat anti-Rabbit IgG Secondary Antibody (1:2000 in TBS-T) (Licor Biosciences, USA) at RT for 1 h on a shaker. Protein band intensities were measured using a Li-Cor Odyssey CLx infrared detection system (LICOR Biosciences) following the manufacturer’s instructions.

### Ratiometric image analysis

Ratiometric analysis was performed using ImageJ software (v.3.91, National Institutes of Health, Bethesda, Maryland, USA). Briefly, Dnmts (green) and DNA (blue) channels of confocal images were separated, and the green one, which were later used for signal measurement, were converted to 32 bit images. Images obtained from Li-Cor Odyssey CLx infrared detection system were converted to 32 bit images. Total signal intensities, as gray values from each pixel, were measured from all embryos for microscopic images, and from the region of interest for WB images using the Image Calculation function in ImageJ software (Al-Zubaidi et al. 2021; Uysal et al. 2021). The background signals were used for thresholding.

### Statistical analysis

All experiments were repeated at least in three times. One-way analysis of variance (ANOVA) followed by Dunn’s post hoc test was performed by using SigmaStat for Windows, version 3.5 (Jandel Scientific Corp). For all tests, *P* < 0.05 was considered statistically significant.

## Results

### Effects of antioxidants on Dnmts and global DNA methylation

**Fig. 1:**
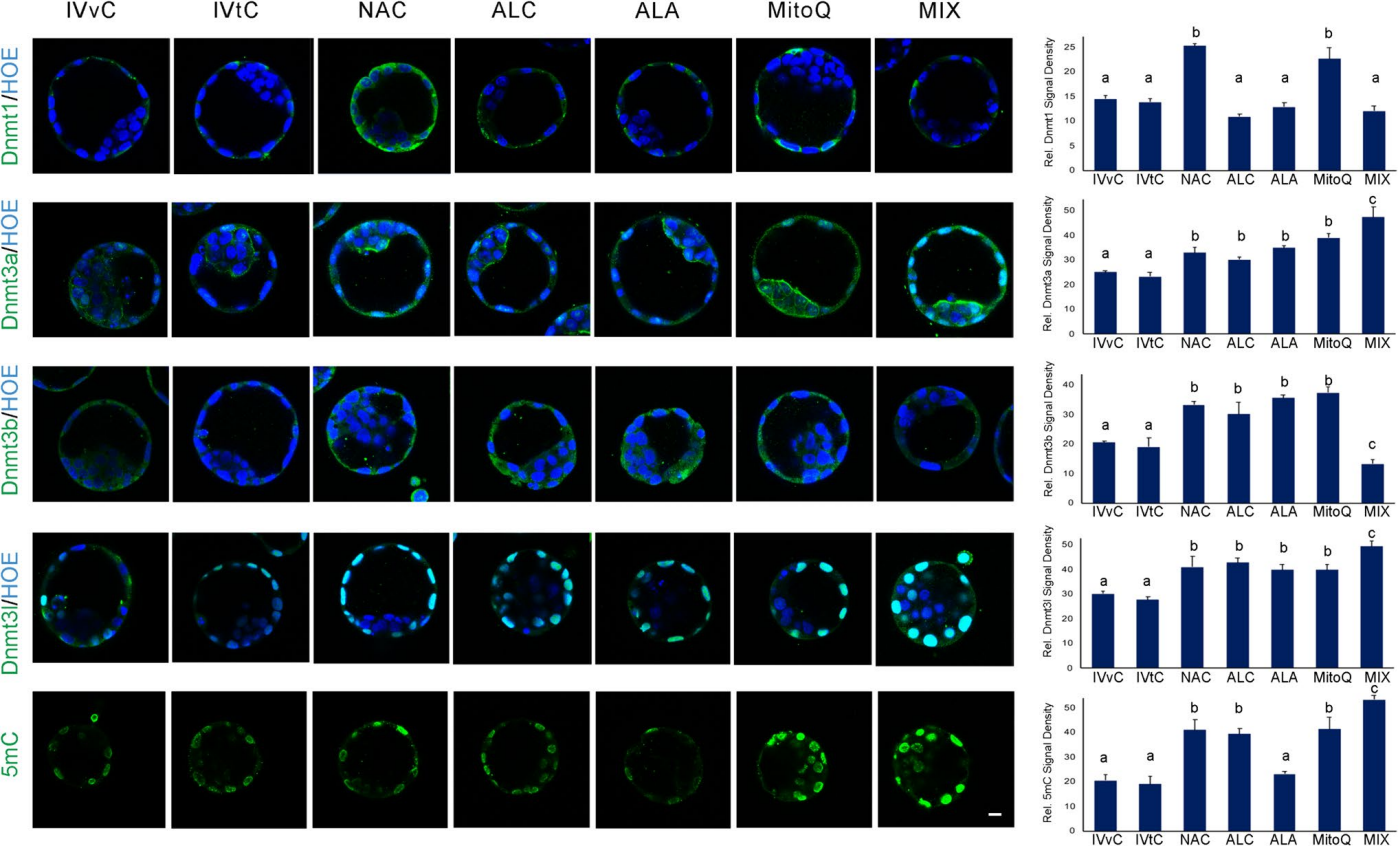
Immunofluorescence analysis of Dnmts and global DNA methylation. The micrographs represent Dnmts and 5mC signals. Bar graphs show the relative staining signal density. In vivo control group (IVvC), in vitro control (IVtC), NAC-, ALC-, ALA-, MitoQ-, and mixture of antioxidants (MIX)-treated embryos. Bars in graphs are represented as mean ± SD. *P* < 0.05 was considered statistically significant, and significant differences between the groups are shown in different letters on the columns. Scale bar: 20 µm

Dnmt1 mainly localized in the trophectoderm (TE) as diffuse cytoplasmic foci, excepting the cell nuclei in all groups (Fig. 1). Dnmt3a signals were primarily observed in cell nuclei and diffusely in the cytoplasm in both embryoblast and TE across all groups (Fig. 1). Dnmt3b staining displayed a diffuse pattern in the cytoplasm without any specific localization to a particular cell population (Fig. 1). Dnmt3l was exclusively confined to the cell nuclei, primarily in TE cells (Fig. 1).

Global DNA methylation status was evaluated with 5mC staining and we found that 5mC signals were located at cell nuclei, more abundantly in the TE (Fig. 1).

**Fig. 2:**
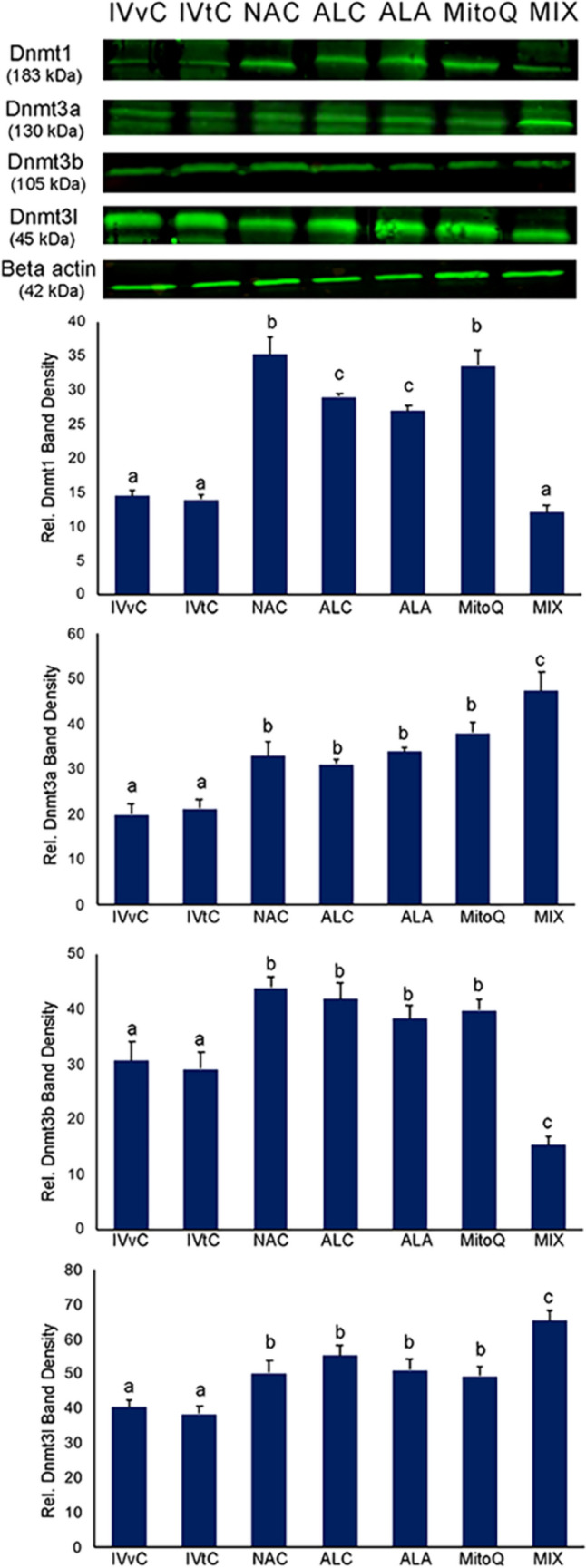
Expression of Dnmt1, Dnmt3a, Dnmt3b, and Dnmt3l in antioxidant-treated embryos. WB assays show the fluorescent-labeled bands specific to Dnmt1, Dnmt3a, Dnmt3b, Dnmt3l, and ß-actin, which was used as the internal control. Bar graphs demonstrates the relative protein expression in the groups. In vivo control group (IVvC), in vitro control (IVtC), NAC-, ALC-, ALA-, MitoQ-, and mixture of antioxidants (MIX)-treated embryos. The different letters on the columns depict statistically significant differences (*P* < 0.05) between the groups. Bars in graphs show mean ± SD

IF and WB consistently revealed that Dnmt1, Dnmt3a, Dnmt3b, and Dnmt3l levels and global DNA methylation levels were comparable in the in vivo and in vitro control groups (Figs. 1, 2).

NAC, ALC, and MitoQ significantly increased the levels of all Dnmts and global methylation (Figs. 1, 2). ALA significantly induced all Dnmts, whereas global methylation did not show any difference. In the mixture AO application group, Dnmt1 levels were comparable, whereas increased Dnmt3a and Dnmt3l levels and decreased Dnmt3b levels were observed. A significant increase in global DNA methylation was detected after mixture AO application.

### Effects of antioxidants on cell differentiation

**Fig. 3:**
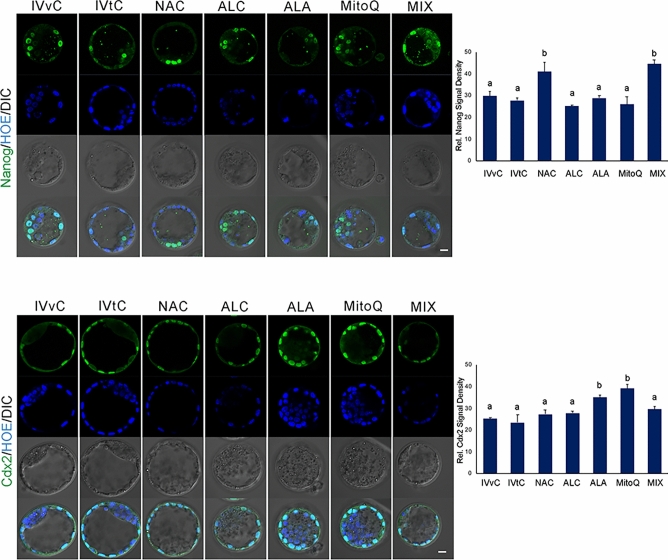
Immunofluorescence analysis of Nanog and Cdx2 after antioxidant exposure. The micrographs represent Nanog and Cdx2 signals. Bar graphs show the relative staining signal density. In vivo control group (IVvC), in vitro control (IvtC), NAC-, ALC-, ALA-, MitoQ-, and mixture of antioxidants (MIX)-treated embryos. Bars in graphs are represented as mean ± SD. *P* < 0.05 was considered statistically significant between the groups, shown as different letters on the columns. Scale bar: 20 µm

To analyze the embryoblast and trophectoderm cell lineage differentiation, levels of Nanog for embryoblast and Cdx2 for trophectoderm were evaluated (Fig. 3). Notably, NAC and mixture AO applications significantly elevated Nanog levels, distinguishing them from the other groups, which exhibited similar levels. Conversely, ALA and MitoQ led to an increase in Cdx2 levels, setting them apart from the other groups, which demonstrated similar outcomes.

### ROS levels

**Fig. 4:**
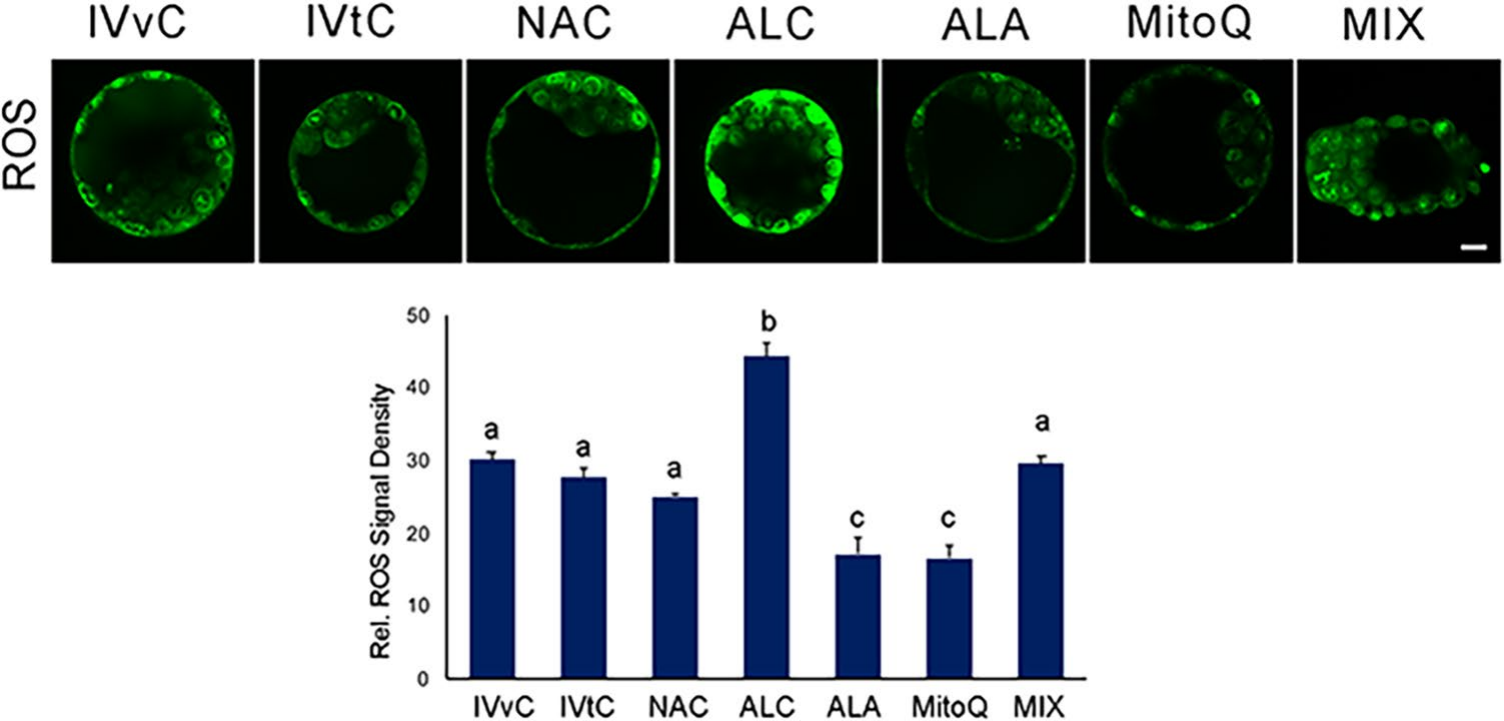
ROS levels after antioxidant exposure. The micrographs represent intracellular ROS signals. Bar graphs show the relative staining signal density. In vivo control group (IVvC), in vitro control (IVtC), NAC-, ALC-, ALA-, MitoQ-, and mixture of antioxidants (MIX)-treated embryos. Bars in graphs are represented as mean ± SD. *P* < 0.05 was considered statistically significant between the groups and are shown as different letters on the columns. Scale bar: 20 µm

ALA and MitoQ application significantly decreased intracellular ROS levels, while in vivo control, in vitro control, NAC, and mixture groups were found to be similar (Fig. 4). Interestingly, ALC application increased the ROS levels.

### TUNEL analysis

**Fig. 5:**
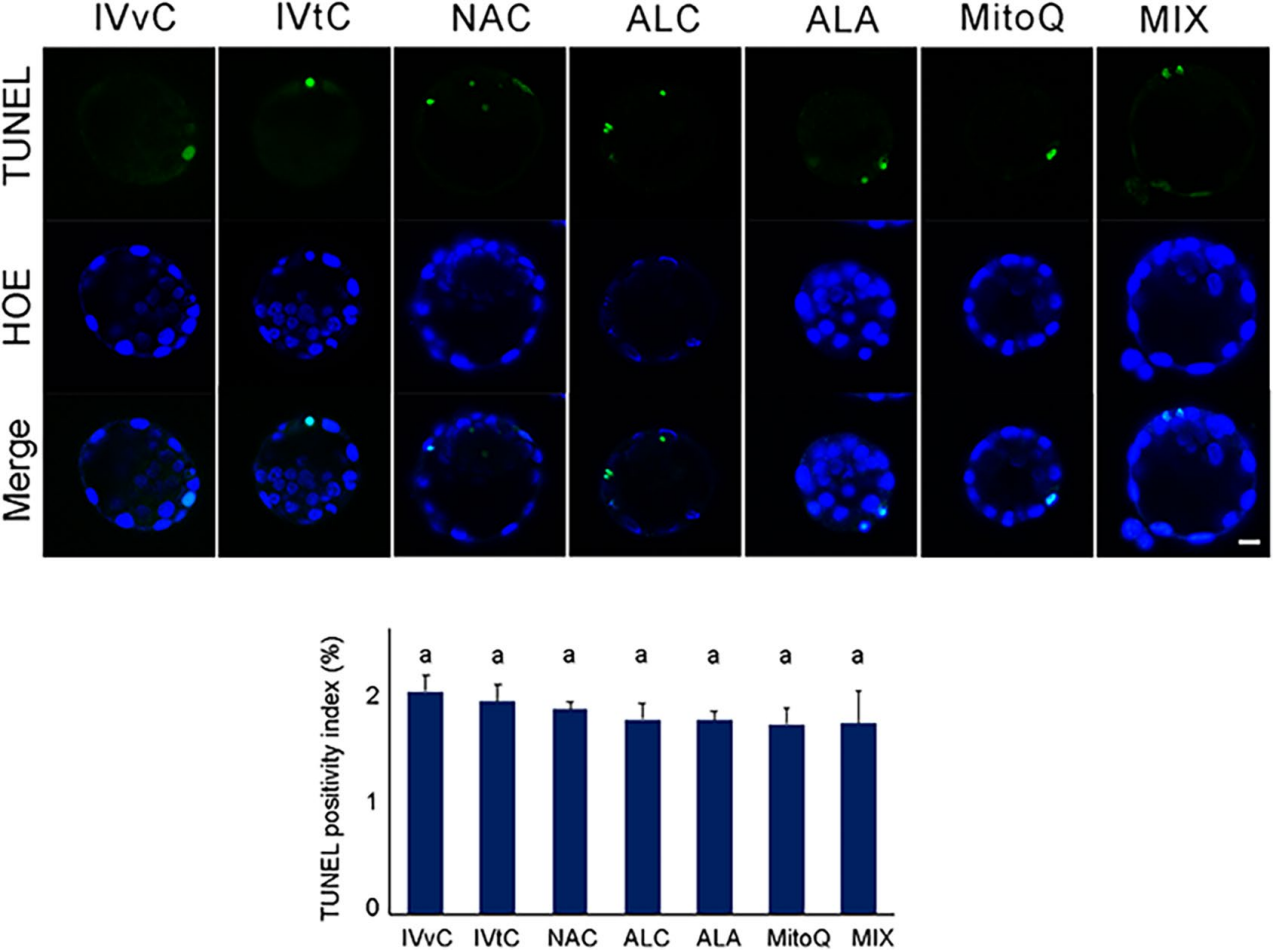
TUNEL assay and positivity index after antioxidant exposure. The micrographs represent TUNEL signals. Bar graphs show TUNEL positivity index. In vivo control group (IVvC), in vitro control (IVtC), NAC-, ALC-, ALA-, MitoQ-, and mixture of antioxidants (MIX)-treated embryos. Bars in graphs are represented as mean ± SD. The different letters on the columns depict statistically significant differences (*P* < 0.05) between the groups. Scale bar: 20 µm

To evaluate cell death score after AO exposure, the TUNEL assay was applied and then the TUNEL positivity index was calculated (Fig. 5). The results revealed that none of the antioxidant treatment caused a difference between the groups.

## Discussion

The beneficial effects of a low-oxygen environment on in vitro embryo development have been shown in many studies (Kasterstein et al. 2013); however, atmospheric oxygen (20%) consumption is still very common in IVF clinics during in vitro embryo culture. Many reports indicate that not only high oxygen levels, but also advanced maternal age causes oxidative stress and, consequently, oocyte maturation and embryo development are negatively affected by these conditions (Ra et al. 2023). Therefore, antioxidant supplementation in embryo culture media seems to be a promising tool to balancing the harmful effects of oxidative stress (Truong and Gardner 2017).

Among the various epigenetic mechanisms, DNA methylation mainly plays critical roles in regulating the expression of development-related genes responsible for early embryo development (Breton-Larrivée et al. 2019). It is known that gradually reduced Dnmt gene expression from zygote to morula stage embryos occurs, and global DNA methylation reaches high levels in blastocysts, the stage at which new genomic imprints are established by de novo methylation processes, and then the maintenance of established methylated epitopes is under the control of Dnmts (Gamage et al. 2018).

The effects of in vitro fertilization procedures, including embryo culture systems, on epigenetic mechanisms need attention. Recently, we demonstrated that Dnmt1, Dnmt3a, and global DNA methylation levels were significantly low in embryos cultured in sequential culture media compared with control and compound embryo culture media (Uysal et al. 2021). Bomfim el al. demonstrated that Dnmt1, 3a, and 3b, and global DNA methylation levels were significantly low in embryos cultured in low oxygen (5%) compared with ones cultured in high oxygen levels (20%) (Bomfim et al. 2017). In the current study, we analyzed the effects of antioxidants containing embryo culture media on DNMTs and global DNA methylation, embryo/trophoblast cell differentiation, ROS levels, and apoptosis during in vitro mouse preimplantation embryo development.

Our results showed that Dnmt1, 3a, 3b, and 3l levels and global DNA methylation levels were comparable between in vivo and in vitro developed embryos. NAC application significantly increased all Dnmts and global DNA methylation levels. Similarly, Yuan et al. documented a similar NAC effect, and they found that in vivo NAC treatment resulted in a significant increase in global DNA methylation and the levels of DNMTs compared with control group in the testes of *Gobiocypris rarus* (Yuan et al. 2019*)*. Heidari et al. showed that the freeze–thaw process of buck sperm reduces DNA methylation, and ALC supplementation may preserve the reduced DNA methylation (Heidari et al. 2022). We found that ALC treatment induced Dnmt and global DNA methylation levels, whereas ALA exposure increased Dnmts levels but did not alter global DNA methylation. Dinicola et al. showed that treating SK-N-BE cells with ALA induces hypermethylation of IL-1β and IL-6, which are modulated by epigenetic mechanisms (Dinicola et al. 2017). It was also documented that ALA reverses 1,4-benzoquinone, a toxic metabolite of benzene, and induced STAT3 hypomethylation in AHH-1 cells (Yang et al. 2015).

The trophectoderm develops placenta, while the embryoblast is essential for a successful embryo development and live birth (Zhao et al. 2018). We found that NAC and mixture AO significantly induced Nanog expression and therefore embryoblast development. On the other hand, ALA and MitoQ increased Cdx2 signals, a sign of trophoblast development. Similarly, He et al. demonstrated that treatment of parthenogenetically developed embryos with 25 μM of ALA increased Cdx2 levels (He et al. 2021). Zhang et al. showed that alpha lipoamide, a derivative of lipoic acid, increased Cdx2 levels and exerted a renal protective effect in a type 2 diabetes mellitus mouse model (Zhang et al. 2023). It has been shown that ALA decreased Nanog levels via the Akt signal pathway in human non-small-cell lung cancer-derived H460 cells in a dose-dependent manner (Phiboonchaiyanan and Chanvorachote 2017), and this was assumed to be a reversive effect of ALA on cancer cells. However, we found that ALA did not alter Nanog levels in blastocysts. This discrepancy can be caused by the type of the cells studied; cancer versus blastocysts.

NAC and mixture AO treatment did not alter ROS levels, whereas ALA and MitoQ significantly inhibited ROS. Interestingly, we found that ALC supplementation significantly induced ROS levels, whereas some studies have demonstrated that ALC decreases ROS levels in oocytes and embryos (Xu et al. 2018, 2020; Shafiei et al. 2020; Mishra et al. 2016). The difference between these works and our study can be explained by the dose differences of ALC, as the tested dose of ALC in these studies were in a range between 1.8 mM and 10 mM, whereas we used 10 μM of ALC. On the other hand, some studies revealed similar ALC effects on ROS levels as our results. Gu et al. found that ALC treatment of zebrafish embryos resulted in increased ATP and ROS levels (Gu et al. 2021). Robinson et al. showed that ketamine dose-dependently attenuated ROS levels in zebrafish larvae in vivo, and that ALC as a dietary supplement dose-dependently increased ROS levels in vivo. In addition, ALC prevents ketamine-induced attenuation of ROS generation in vivo (Robinson et al. 2019).

In conclusion, the current study demonstrates that antioxidants acting via different physiological pathways differentially affect Dnmts, global DNA methylation, cell differentiation, and oxidative stress. Further studies such as gene sequencing are needed to clarify which pathways are affected and are thus necessary for these processes. Although this point could be a limitation for the present manuscript, our results are fundamental to show the effects of antioxidants on DNA methylation.

## Data Availability

The data that support the findings of this study are available from the corresponding author upon reasonable request.
